# Positive relationship between species richness and aboveground biomass across forest strata in a primary *Pinus kesiya* forest

**DOI:** 10.1038/s41598-018-20165-y

**Published:** 2018-02-02

**Authors:** Shuaifeng Li, Jianrong Su, Xuedong Lang, Wande Liu, Guanglong Ou

**Affiliations:** 1 Research Institute of Resource Insects, Chinese Academy of Forestry, Kunming, 650224 China; 2 Pu’er Forest Ecosystem Research Station, China’s State Forestry Administration, Kunming, 650224 China; 3 Key laboratory of State Forest Administration on Biodiversity Conservation in Southwest China, Southwest Forestry University, Kunming, 650224 China

## Abstract

Both biodiversity and biomass are important variables in forest ecosystems, and the relationship between them is critical for ecosystem functioning and management. The primary *Pinus kesiya* forest is increasingly threatened by human disturbance in Yunnan Province. We observed that species richness had a positive impact on aboveground biomass across all forest vegetation layers, and this relationship was strongest in the herb layer. The asymptotic relationship between cumulative species number and aboveground biomass suggested that individual of *Pinus kesiya* trees with relatively large diameters contributed the majority of the aboveground biomass in the tall tree strata due to their strong competitive advantage over other tree species. Although aboveground biomass increased with stand age in the tall tree strata, climate factors and the soil nutrient regime affected the magnitude of the diversity-productivity relationship. Stand age had no significant effect on species richness and aboveground biomass in the forest understory. The effect of the positive diversity-productivity relationship of the tall trees on the shrub layer was negligible; the diversity-productivity relationship in the forest understory was significantly affected by the tall tree aboveground biomass. The tall trees have increased the strength of the positive diversity-productivity relationship in the forest understory.

## Introduction

The relationship between biological diversity and ecosystem functioning has stimulated research on the relationship between terrestrial plant species richness and temporal variation in biomass production during the past two decades^1,2^. A clear forest biodiversity-biomass relationship has been shown in previous studies. Positive species diversity and biomass relationships are ubiquitous in most forest ecosystems around the world, and species loss in these ecosystems negatively impacts ecosystem functioning^3,4^. Most previous research has focused on the relationship between species diversity and productivity, not biomass, in forest ecosystems^5,6^. However, biomass is strongly correlated with productivity when the effects of stand age are taken into account^4,7,8^. An increasing number of studies have focused on the relationship between biodiversity and aboveground biomass, as well as the mechanisms resulting in variations in both species functional traits and environmental conditions in tropical, temperate and boreal forest ecosystems^4,9^.

Previous studies have shown that tree species in the overstory play important roles in mediating the effects of environmental conditions and disturbance on understory species richness^10,11^. Accordingly, studies on the relationships between species richness and biomass across forest strata have been carried out in boreal and subtropical forests^9,11–14^. Reich *et al*. confirmed that a positive species richness and aboveground biomass relationship across forest strata was found, and the understory species richness is mediated by the indirect effects of the dominant producers on resource availability and heterogeneity in a boreal forest^13^. Further evidence from Canada’s National Forest Inventory studies shows that positive relationships between species richness and aboveground biomass in understory vegetation may not affect overstory tree species^12^. Simultaneously, similar findings have also indicated that diversity-productivity relationships in the forest understory were not associated or were negatively associated with the aboveground biomass of the overstory layer^7^. In contrast, the species diversity of the overstory layer significantly increased the species diversity of the understory layer in a subtropical forest, while the effect of the aboveground biomass of the overstory on the diversity and aboveground biomass of the understory was negligible^14^. The occurrence of shade species from the canopy trees in the understory layer is a common phenomenon, and the overstory-understory species richness relationship suggests that different functional groups have specific growth strategies^11^. There is no doubt that the size of the overstory, the interaction between species richness and aboveground biomass in this layer, and its indirect effects on resource availability and heterogeneity mediate the relationship between species richness and aboveground biomass in the understory layers^7,12,13^.

The driving mechanisms of comprehensive causal relationships could be explained by both selection and complementarity effects^15^. A previous study demonstrated that the majority of productivity of the forest ecosystem occurred in the overstory, while understory plants have relatively low contributions to the total biomass stock^16^. The relationship between species richness and biomass is more complex in natural ecosystems because of species dominance and composition^17^. The selection effect suggests that species with the most productive traits will have greater opportunities to dominate the biomass of species-rich polycultures through interspecific competition, while the complementarity effect suggests that species can capture resources to maintain diversity as a result of niche partitioning or interspecific interactions when they occur together in plant communities^15,18^.

Although positive species diversity and aboveground biomass relationships are mostly found across forest strata that exhibit higher species richness and many trophic levels^2,7^, biotic and abiotic factors affect the magnitude and quality of such relationships, and these effects should be considered, especially by increasing the range of variables included in multivariate studies, such as competition intensity, resource heterogeneity and population dynamics^13^. Stand age is closely associated with the species richness-biomass relationship^9^. Simultaneously, the consideration of environmental conditions may be crucial for understanding the relationship between species richness and biomass along with two drivers^4^: First, higher resource availability supports more biomass accumulation under more favourable climate and soil nutrient conditions^15^. Second, the diversity-productivity relationship shows a unimodal association from harsh to favourable climate and soil nutrient conditions^4^. The extents of the influences of climate and local site conditions on plant species diversity differ significantly among vegetation strata^19^. Climate, soil nutrient conditions and stand age are recognized as directly or indirectly affecting the species richness-aboveground biomass relationship, but were rarely explicitly considered because of the instability in environmental conditions^9,12^.

Primary *Pinus kesiya* forests have their largest distribution throughout northern India, the Philippines, Myanmar, Vietnam, Laos, Thailand and Yunnan Province, China^20,21^. In Yunnan Province, China, primary *Pinus kesiya* forests play an important role in local forest ecosystem functioning, including carbon sequestration and biological conservation, in addition to timber production and resin tapping. Deforestation has decreased the area of forest over recent decades. Degradation of the remaining stands and climate change have affected the regeneration, growth and distribution of primary *Pinus kesiya* forests, increasing species loss and threatening stable ecosystem functioning^22^. *Pinus kesiya* usually invades monsoon evergreen broadleaf forest areas after the broadleaf species have been cleared^23^, causing the vertical structure of the community to experience obvious species stratification. There is a distinct difference between a primary *Pinus kesiya* forest and a boreal or subtropical evergreen broadleaf forest, as that almost all *Pinus kesiya* individuals are distributed in the overstory layer in the primary *Pinus kesiya* forest, while most of the broadleaf species occupy the understory layer. There is little information regarding the relationships between the species richness and aboveground biomass across forest vegetation strata in primary *Pinus kesiya* forests.

In this study, we used a multivariate model to evaluate path hypotheses to assess the relationship between the species richness and aboveground biomass of the tall tree, short tree, shrub, herb and liana layers in a primary *Pinus kesiya* forest. We also analysed the influences of the tall tree layer, stand age, soil nutrient regime and climate factors as mechanisms driving the relationship between species richness and aboveground biomass across forest vegetation strata based on existing theoretical frameworks^7^. Based on the existing study of Zhang *et al*.^12^ and our field data, we summarized theoretical frameworks to test the following paths: (1) the relationships between species richness and aboveground biomass across forest vegetation strata, including the tall tree, short tree, shrub, liana and herb layers; (2) the influences of the soil nutrient regime, stand age and climate factors on the species richness across forest vegetation strata and the effect of stand age on aboveground biomass; (3) the influences of species richness and aboveground biomass in the tall tree strata on the paths of (1) and (2) in the short tree, shrub, liana and herb layers; and (4) the influence of species richness in the short tree strata on aboveground biomass across all layers. The aim of this study was to disentangle the potential mechanisms that mediate the relationship between species richness and aboveground biomass across forest strata and to provide a reference for the management of natural subtropical coniferous forests under global climate change.

## Results

### Relationships between species richness and aboveground biomass across all forest vegetation strata

**Figure 1:**
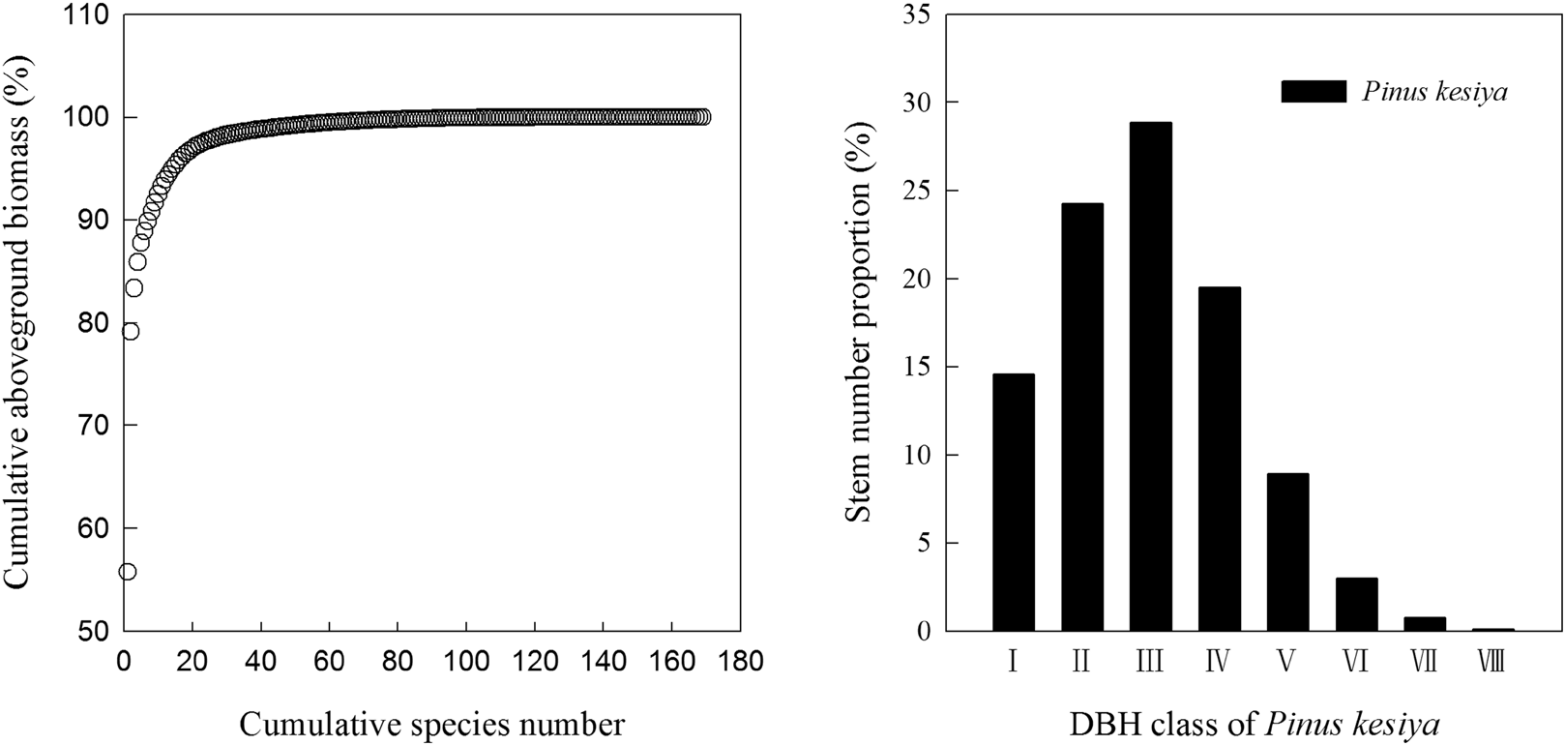
Cumulative aboveground biomass was ranked against cumulative species number and DBH class distribution of *Pinus kesiya* at the community level. DBH, diameter at breast height; I, DBH < 9 cm; II, 9 ≤ DBH < 20 cm; III, 20 ≤ DBH < 30 cm; IV, 30 ≤ DBH < 40 cm; V, 40 ≤ DBH < 50 cm; VI, 50 ≤ DBH < 60 cm; VII, 60 ≤ DBH < 70 cm; VIII, 70 ≤ DBH < 80 cm.

Few species contributed a great deal to the aboveground biomass in the primary *Pinus kesiya* forest. The cumulative proportion of aboveground biomass showed that there was a strong asymptotic relationship with the cumulative species number (Fig. 1a). One of 169 species (accounting for 83.25% of the species number in all plots) was found to have the contribution of more than 50% of the total aboveground biomass, up to 55.89% for *Pinus kesiya*, while most tree species contributed little. The majority of *Pinus kesiya* stems were in the tall tree layer, up to 85.43% (Fig. 1b).

**Figure 2:**
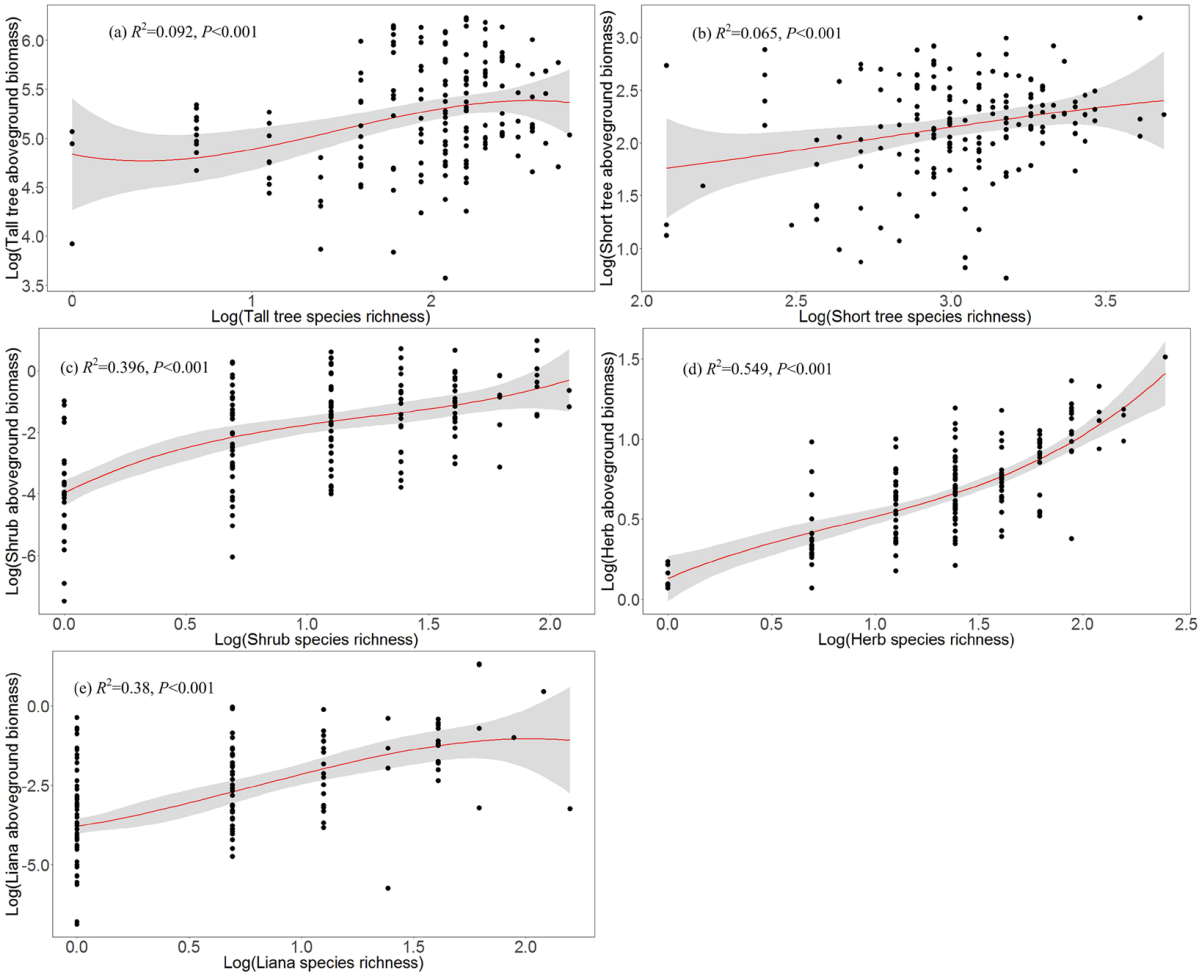
Relationships between the tall tree species richness and aboveground biomass, short tree species richness and aboveground biomass, shrub species richness and aboveground biomass, herb species richness and aboveground biomass, and liana species richness and aboveground biomass. The red fitted lines are from the OLS polynomial regression, and the shaded areas show the 95% confidence intervals for the fit.

The aboveground biomass values of the tall tree, short tree, shrub, herb and liana layers were positively correlated with the species richness of the tall tree, short tree, shrub, herb and liana layers based on the cubic OLS regression, respectively (Fig. 2a–e). The aboveground biomass linearly increased with the species richness across the forest vegetation strata. The relationship between species richness and aboveground biomass for the tall tree layer was weaker than those for the shrub, herb and liana layers. The aboveground biomass and species richness in the herb layer showed a stronger positive correlation than those in the other forest vegetation strata (Fig. 2d).

### Effects of species richness, stand age and abiotic factors on aboveground biomass in the tall tree strata

**Figure 3:**
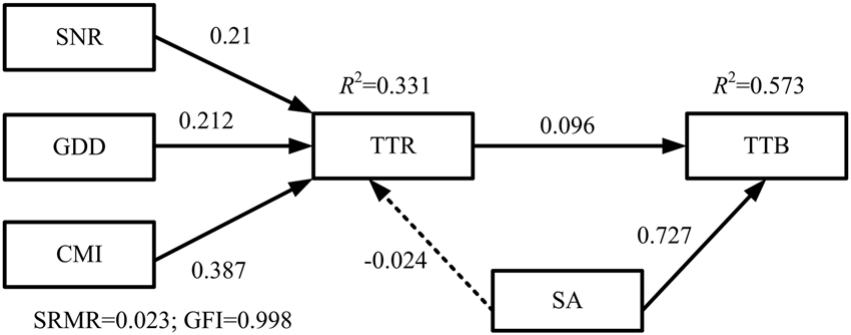
Structural equation models linking tall tree aboveground biomass (TTB) and tall tree species richness (TTR) with the influences of climate, soil, and stand age factors. Solid lines indicate significant paths (*P* < 0.05), and dashed lines indicate non-significant paths (*P* ≥ 0.05). TTB, tall tree biomass; STR, short tree richness; SNR, soil nutrient regime; GDD, growing degree days; CMI, climate moisture index; SA, stand age; SRMR, standardized root mean square residual; GFI, goodness-of-fit index.

The structural equation model linking the aboveground biomass and species richness of the tall tree layer showed a good fit (SRMR = 0.023, GFI = 0.998), accompanied by variations in the tall tree species richness and stand age of up to 57.3%. Our results suggested that the tall tree species richness and stand age directly affected the aboveground biomass of the tall tree strata (*r* = 0.096 and *r* = 0.727, respectively) (Fig. 3). Simultaneously, there were positive relationships among the soil nutrient regime, growing degree days, climate moisture index and tall tree species richness, of which the soil nutrient regime indirectly affected the aboveground biomass of the tall tree layer through the tall tree species richness (*r* = 0.027) (Fig. 3). Stand age had a stronger effect than species richness on the tall tree aboveground biomass based on the SEM results, while the tall tree species richness was not influenced by stand age (*r* = −0.024).

### Effects of the tall tree strata on the species richness and aboveground relationship in the forest understory layer

**Figure 4:**
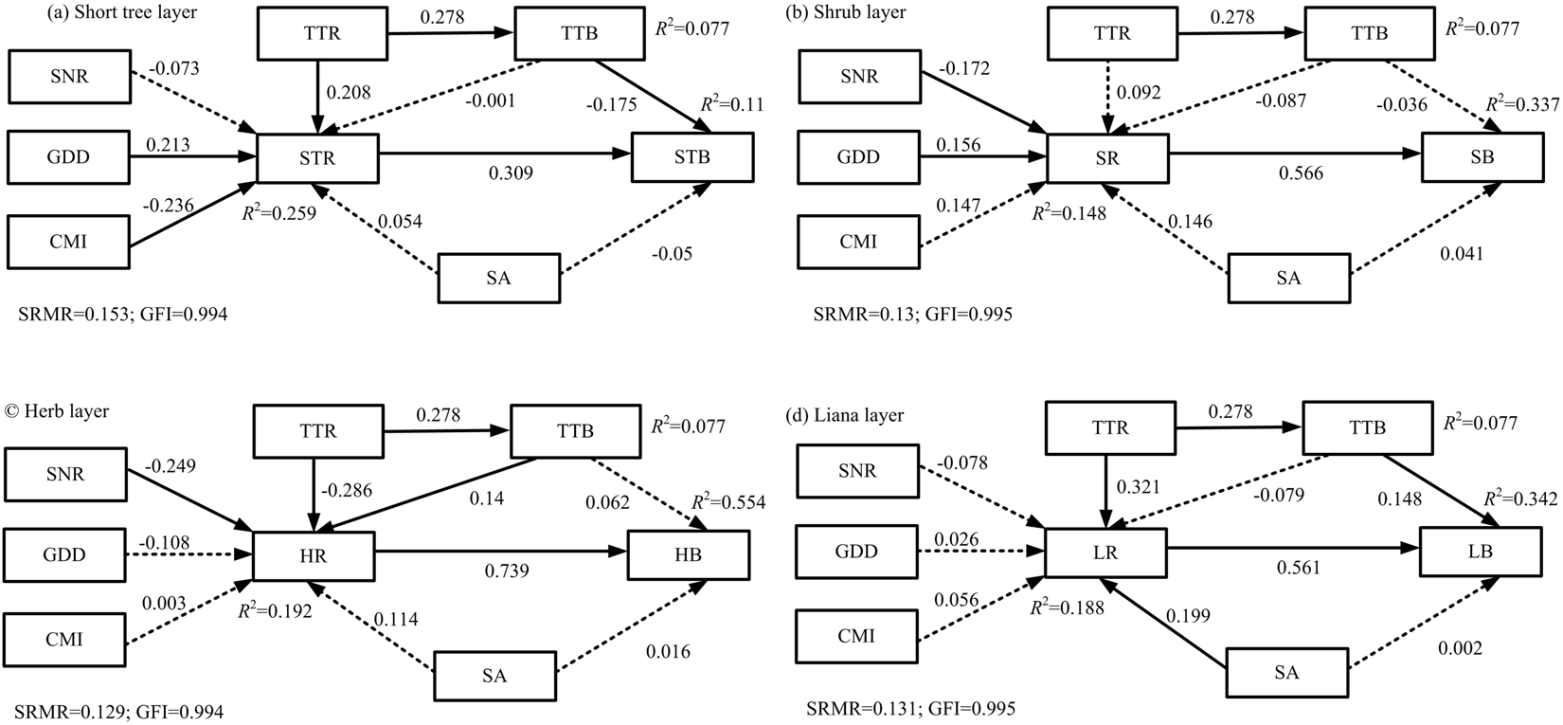
Structural equation models linking aboveground biomass and species richness across forest strata, including the short tree (**a**), shrub (**b**), herb(**c**) and liana (**d**) layers. STR, SR, HR and LR represent the species richness of the short tree, shrub, herb and liana layers, respectively. STB, SB, HB and LB represent the aboveground biomass of the short tree, shrub, herb and liana layers, respectively. The other abbreviations are explained in Fig. 3.

The structural equation models showed good fits with the data from the forest understory layer (Fig. 4a–d). The species richness of the forest understory layer had a direct positive effect on the corresponding aboveground biomass. Stand age had no significant relationship with species richness or aboveground biomass in the short tree, shrub and herb layers (Fig. 4a–c). The tall tree species richness had a positive direct effect on the short tree species richness and indirectly affected the short tree aboveground biomass through the short tree species richness along with the greater species richness in the tall tree layer and the greater short tree aboveground biomass. However, the tall tree species richness had a negative indirect effect on the short tree aboveground biomass through the tall tree aboveground biomass. Growing degree days and the climate moisture index affected the short tree aboveground biomass through the short tree species richness. The short tree aboveground biomass increased with greater growing degree days but decreased with climate moisture index.

The soil nutrient regime and growing degree days affected the shrub aboveground biomass through the shrub species richness (Fig. 4b). The soil nutrient regime, tall tree aboveground biomass and species richness affected the herb aboveground biomass through the herb species richness, but the soil nutrient regime had an indirect negative effect on the shrub aboveground biomass (Fig. 4c). The tall tree aboveground biomass showed a direct positive correlation with the aboveground biomass of the liana layer, while the tall tree species richness indirectly affected the liana aboveground biomass through the liana species richness and tall tree biomass. In addition, stand age affected the liana aboveground biomass through the liana species richness (Fig. 4d). The climate moisture index showed no influence on the aboveground biomass in the shrub, herb or liana layers, while growing degree days had no influence on the aboveground biomass in the herb and liana layers.

## Discussion

Our findings show that the positive relationship between species richness and aboveground biomass is ubiquitous across forest strata, and widely exists in all forest vegetation strata in primary *Pinus kesiya* forests. Such relationships are consistent with previous studies that have found a positive association between species richness and aboveground biomass in most forest ecosystem^5,7,12^. Furthermore, we provide more evidence for this relationship across forest vegetation strata, especially in subtropical pine-oak forests. These results differ from those found in a study by Ali and Yan, which indicated that the tall tree layer had negligible effects on the short tree layer in terms of aboveground biomass^14^, while our results show that the tall tree aboveground biomass had a negative effect on the short tree aboveground biomass in the primary *Pinus kesiya* forest ecosystem, which covers a large area of the subtropical zone in Yunnan Province, SW China. The reason for these findings may be that distinguishing between the tall tree and short tree vegetation strata may be more complex and more difficult in evergreen broadleaf forests without the presence of productive pines along with the weakening of niche partitioning in the vertical spaces of the forest.

We observed that direct and indirect path processes mediated the relationships between species richness and aboveground biomass across the forest vegetation strata in a primary *Pinus kesiya* forest. As a rule, existing studies have demonstrated that the species richness of the tall tree strata was dependent on the macroclimate at a relatively large scale, which could increase the abundance of dominant species and enhance light utilization efficiency in the tall tree strata^13,16^. *Pinus kesiya* has been described as a species with relatively high production due to its prolonged photosynthetic activity and high nutrient uptake through the rapid turnover of nutrients in subtropical pine-oak forests^21^. This species is often considered to be the strongest competitor and has many large diameter individuals; it is therefore the main contributor to woody biomass production in these forests, which dominates the relationship between species richness and aboveground biomass in the tall tree layer. Larger trees of *Pinus kesiya* have a greater contribution to the aboveground biomass than smaller diameter trees in old-growth forests^21^. Although specific driving mechanisms were not analysed in our study, the role of *Pinus kesiya* could be potentially explained by the selection effect^15,18^. This species may have been the most productive species in the local species pools at the beginning of the establishment of the forest community and gradually became the most dominant species in the tall tree strata, which negatively affected the growth and regeneration of short tree species^15^. Our results show that the tall tree aboveground biomass had a negative effect on the aboveground biomass of the short tree and shrub layers, which could also be better explained by the amount of light-blocking tissue in the canopy of tall tree species.

The tall tree species richness significantly promoted the aboveground biomass of the short tree and liana layers. The short tree species are predominantly mountainous subtropical broadleaf species^20^, which could prevent strong interspecific competition and promote greater site resource utilization along with tall tree species according to the complementarity effect^7^. The coexistence of short tree species was beneficial in increasing the woody biomass production due to enhanced resource use efficiency by means of water and nutrient uptake facilitation, niche partitioning and the control of herbivores and disease^24^. Short tree species have specific environmental requirements and can maintain the species diversity in primary *Pinus kesiya* forests even if some species exhibit a lower woody biomass production which could also offset the negative effects through niche partitioning. Recent studies have suggested that coexisting species were more functionally similar or redundant in species-rich communities^4^. Our results support this idea that shade-tolerant and evergreen broadleaf species composed the short tree strata and demonstrated more niche overlap. The strength of the relationship between species richness and aboveground biomass in the short tree strata was greater than that in the tall tree layer. Because of the lack of highly productive species in the short tree strata, the short tree species also had a lower contribution to the total aboveground biomass across forest vegetation strata. In addition, the trees of larger diameter are important host trees in subtropical forest ecosystems and are beneficial to the establishment and growth of lianas. The larger trunks of most *Pinus kesiya* individuals and other canopy tree species facilitated climbing by liana species, and therefore, the species richness of the tall tree strata increased with the aboveground biomass production of lianas in accordance with stand age.

Stand age had a positive influence on the tall tree aboveground biomass accounting for 80.31% of the variance. It is widely believed that stand age is a critical driver of individual species dynamics, standing biomass and productivity^9^. Although the net primary productivity of old-growth stands is lower than that in younger stands of primary *Pinus kesiya* forests^21^, it was confirmed that the aboveground biomass increased with stand age in this primary *Pinus kesiya* forest^20^. Our results indicate that stand age had no impact on the aboveground biomass of the short tree, shrub, or herb layers. The magnitude of the positive diversity-productivity relationship in the forest short tree layer was affected by the shade of canopy trees and the environmental conditions^7^.

Despite the relatively low contribution of the shrub and herb layers to the total aboveground biomass, they contributed a substantial proportion of the annual litter fall which contributes to the total annual ecosystem nutrient uptake^25^. Litter fall and fine root decomposition change the supply of N and P and their availability in the soil, resulting in changes in plant growth and biomass production. A previous study showed that lower soil N:P tatios promoted the growth and biomass production of the shrub and herb layers^26^, which is in agreement with our results. However, the N:P ratio had a positive indirect influence on the tall tree aboveground biomass by enhancing plant photosynthesis as a result of greater N availability. In addition, climate factors also affected the species richness of the short tree strata, and environmental heterogeneity had an important effect on the short tree strata species diversity^11^. We observed that the short tree strata accounted for most of the vascular plant species diversity in the primary *Pinus keisya* forest, in contrast to the low tall tree species diversity. Short tree species have specific niches and are better able to adapt to different environmental conditions, which supports the idea that the climate moisture index, growing degree days and the soil nutrient regime affect the aboveground biomass across forest vegetation strata, especially in the tall tree layer^7,12^. Our results are consistent with those from a recent study on the relationship between species richness and biomass from boreal to subtropical forests in China^4^.

## Conclusions

The positive relationship between species richness and aboveground biomass found in our study is similar to those described in previous studies, indicating that this relationship is ubiquitous across forest vegetation strata at large spatial scales. Moreover, our results also show that the relationship in the tall tree strata was weaker than that in the shrub, herb and liana layers. However, the tall tree species richness was positively correlated with the species richness and aboveground biomass of the short tree and liana layers. In contrast, the species richness of the tall tree strata had an indirect negative effect on the aboveground biomass of the herb layer. The species richness and aboveground biomass in the tall tree strata had a complex effect on the biodiversity-biomass relationship across forest vegetation strata in accordance with two important mechanisms of biological maintenance: the selection effect and the complementarity effect. The tall tree species had an obvious resource-filtering effect, which strengthened the intensity and magnitude of the positive biodiversity-biomass relationships in the forest understory strata. Furthermore, climate factors had an important influence on the positive biomass-richness relationship in the tall tree strata. The soil nutrient regime and shading by tall tree species directly or indirectly affected the species richness-biomass relationships in the short tree, shrub, and herb layers. Our study provides new insight into the ecology of the primary *Pinus kesiya* forest, and the results almost did not show an effect of species loss on biomass or productivity because of anthropogenic disturbance. For a better understanding of the biodiversity-biomass relationship, long-term observations will be essential in future studies.

## Materials and Methods

### Study area and site description

The study was conducted in primary *Pinus kesiya* forests that are mainly distributed in the southern and southwestern parts of Yunnan Province, China. This area has mountainous topography, with an altitude ranging from 850 m to 1850 m. This region has a typical monsoon climate with two distinct seasons: a rainy season (from May to October) and a dry season (from November to April). The annual mean temperature ranges from 17 °C to 18.5 °C. The annual precipitation ranges from 1100 mm to 1550 mm, with favourable hydrothermal conditions, and more than 80% of the annual precipitation falls during the rainy season^23^.

*Pinus kesiya* and *Schima wallichii* are the dominant canopy tree species in all the sampled stands. The subcanopy tree species in the forest are *Castanopsis hystrix*, *Castanopsis echidnocarpa*, *Castanopsis calathiformis*, *Castanopsis fleuryi*, *Lithocarpus truncatus*, *Lithocarpus fenestratus*, *Machilus rufipes*, *Betula alnoides*, *Rhus chinensis*, and *Alnus cremastogyne*. The shrub layer is dominated by *Symplocos paniculata*, *Breynia fruticosa*, *Ficus hirta*, *Melastoma affine*, *Glochidion eriocarpum*, and *Inula cappa*. The herb layer is dominated by *Eupatorium adenophorum*, *Zingiber densissimum*, *Hedychium flavum*, *Imperata cylindrica* var. *major*, *Woodwardia japonica*, *Dicranopteris pedata*, *Scleria levis*, and *Dianella ensifolia*. *Mucuna macrocarpa*, *Craspedolobium schochii*, *Embelia ribes*, *Smilax* spp., *Gnetum montanum*, and *Celastrus monospermus* are the dominant liana species in the primary *Pinus keisya* forest.

### Forest inventory data

**Figure 5:**
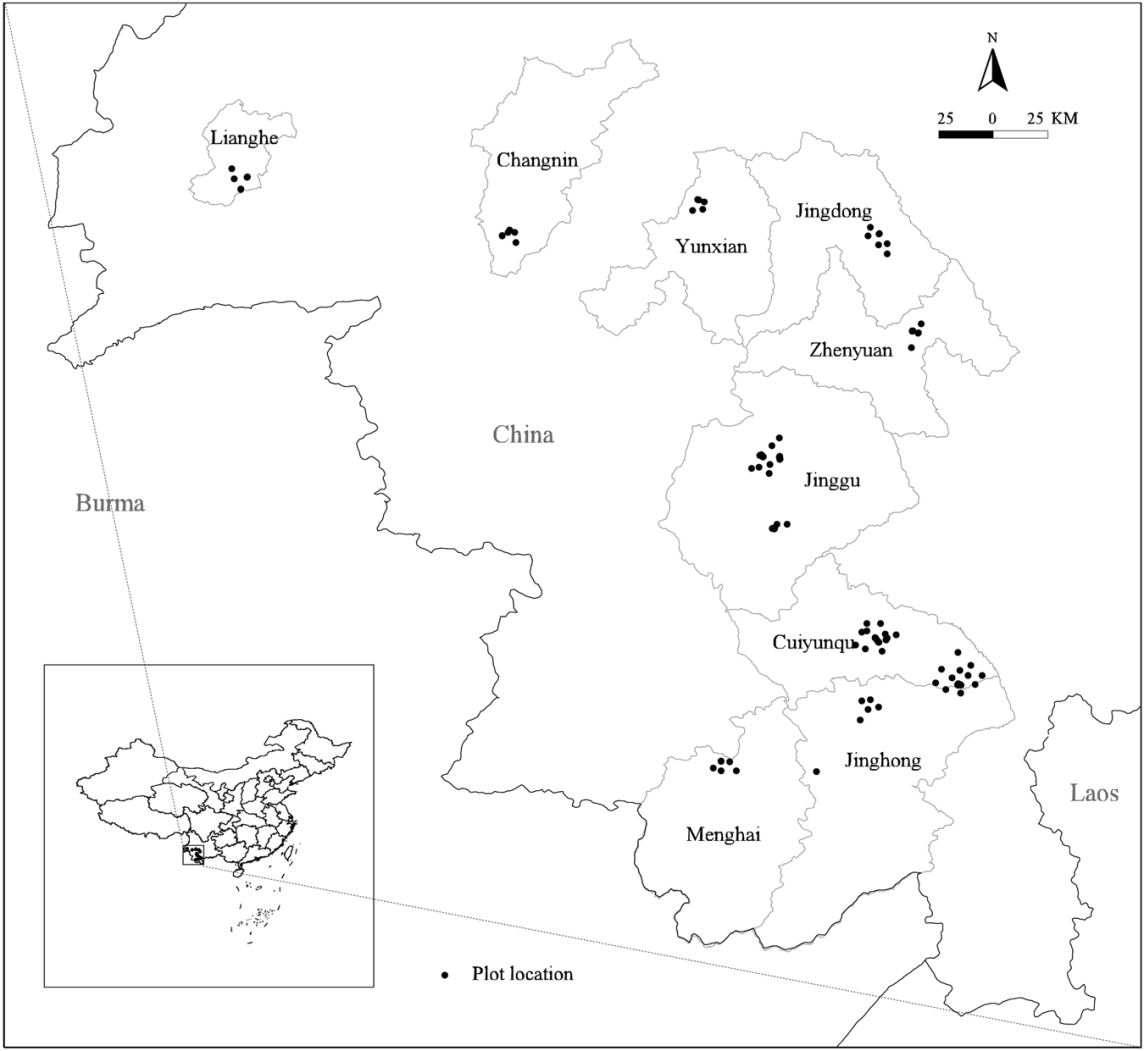
The locations of the plots in the study area. The map was generated using the software ArcGIS 10 (Environmental Systems Research Institute, Inc. Redlands, CA, USA; http://www.esri.com).

The data were from 170 field sampling plots located in nine counties, including Simao, Jinghong, Menghai, Jinggu, Zhenyuan, Jingdong, Yunxian, Changning and Lianghe in southern and southwestern Yunnan Province, China. The plots ranged from 22°11′ to 24°38′ N and from 22°11′ to 24°38′ E. The plots were 20 m × 20 m (400 m^2^) based on the requirement in *Yunnan vegetation*^23^. All plots were measured between 2012 and 2014 (the distribution of plots is shown in Fig. 5). Within each plot, the forest vegetation was classified as belonging to the tall tree, short tree, shrub, herb or liana layers. All trees, lianas and shrubs were measured for their diameter at breast height (DBH) if they had a DBH ≥ 1 cm. Five 2 m × 2 m subplots were used for the survey of the herb layer, including tree seedlings, shrubs and herbaceous plants. Plant species, number, height and environmental factors, including altitude, slope, aspect, and slope position, were recorded. We defined all stems in each plot with DBH ≥ 9 cm as part of the tall tree layer; the short tree layer included all trees with DBH < 9 cm and height ≥1.3 m as well as shrubs ≥1.3 m in height. The shrub layer included all shrubs and trees <1.3 m in height. The liana layer included lianas with stems of all different sizes in the plots. The herb layer included all non-woody vascular plants, such as ferns, graminoids and saprophytes^12^. We used species richness as a species diversity index for the tall tree, short tree, shrub, herb, and liana layers. We recorded a total of 203 trees, 44 shrubs, 102 herbs and 41 lianas in all plots. Stand age (SA) was determined as the mean age of the three largest *Pinus kesiya* individuals. We fitted an age-DBH equation for *Pinus kesiya* using the formula *y* = 3.326*DBH*^0.733^ (*y* is the tree age, *R*^2^ = 0.802, *P* < 0.001, *F* = 357.323, *n* = 90).

### Estimation of aboveground biomass

Aboveground biomass (AGB, Mg·ha^−1^) across forest vegetation strata was estimated as the sum of the stems, branches, and leaves using allometric equations including trees with DBH ≥ 5 cm, shrubs and small trees with DBH < 5 cm, and lianas (DBH ≥ 2 cm) (Table 1). We classified species occurring in the forests into the following 9 plant functional groups and obtained their aboveground biomass using species-specific allometric equations based on DBH, height or length: (1) *Pinus kesiya*^27^, (2) *Schima wallichii* (the allometric equation was built by the authors), (3) *Castanopsis hystrix* and Fagaceae species with similar functional traits^28^, (4) *Betula alnoides*^29^, (5) *Rhus chinensis*^30^, (6) *Alnus cremastogyne*^31^, (7) other mixed tree species for less frequently occurring species^32^, (8) shrubs and small trees^33^, and (9) lianas^34^. We used a destructive harvesting method to measure the aboveground biomass of the herb layer, including herbs, shrubs and tree seedlings (DBH ≤ 1 cm)^20^.

**Table 1:** Allometric biomass equations for different components of *Pinus kesiya* and other broadleaf species. *Y* is the biomass of the tree component (kg), *D* is the diameter at breast height (cm), *H* is the tree height (m) and *L* is the liana length (m).

Number	Tree species/group	Component	Allometric equation
1	*Pinus kesiya*	Trunk	*Y* = 0.0808*D*^2.5374^
		Branch	*Y* = 0.0007*D*^3.4663^
		Needle	*Y* = 0.0015*D*^2.504^
2	*Schima wallichii*	Aboveground	*Y* = 0.24*D*^2.072^
3	*Castanopsis hystrix*	Trunk	*Y* = 0.06411*(D*^2^*H)*^0.8699^
		Bark	*Y* = 0.0105*(D*^2^*H)*^0.8246^
		Branch	*Y* = 0.00011*(D*^2^*H)*^1.3949^
		Leaf	*Y* = *0.00000*2*8(D*^*2*^*H)*^1.6052^
4	*Betula alnoides*	Trunk	*Y* = *0.15D*^2.1969^
		Branch	*Y* = *0.0313D*^2.2118^
		Leaf	*Y* = *0.0094D*^2.0184^
5	*Rhus chinensis*	Aboveground	*Y* = *0.3D*^2.077^
6	*Alnus cremastogyne*	Trunk	*Y* = 0.027388(*D*^2^*H*)^0.898869^
		Bark	*Y* = 0.012101(*D*^2^*H*)^0.854295^
		Branch	*Y* = 0.014972(*D*^2^*H*)^0.875639^
		Leaf	*Y* = 0.010593(*D*^2^*H*)^0.813953^
7	Other mixed trees	Aboveground	*Y* = *0.1381D*^2.3771^
8	Lianas	Aboveground	*Y* = 0.074*(*D*^2^*L)^0.8495^
9	shrubs and small trees	Aboveground	LN(*Y*) = −3.5 + 1.65*LN(*D*) + 0.842*LN(*H*)

### Environmental factors

The soil nutrient regime (SNR), growing degree days (GDD, °C) and climate moisture index (CMI, mm, annual precipitation minus annual potential evapotranspiration) were used to account for the effect of environmental conditions and stand growth on aboveground biomass. We used the N:P ratio as a measure of the soil nutrient regime to represent local site conditions. Climate data were obtained from the Climate AP software and have been revised for use in the Asia-Pacific region^35^. The GDD and CMI values represented the overall energy available for plant growth and water availability for plants^7^.

### Data analysis

We regressed the species richness on aboveground biomass across forest vegetation strata to assess the relationships between these two variables using ordinary least squares (OLS) regressions with a cubic smoothing spline. The cumulative value of aboveground biomass was plotted against the cumulative species number (these species represent 99.53% of the total biomass in the landscape) according to the magnitude of the aboveground biomass of each species in all plots, which could indicate the relative contribution of the species to the aboveground biomass^17^. We used a structural equation model (SEM) in our analyses to determine the effects of response variables, which were related to the tall tree and environmental conditions on the relationships between the species richness and aboveground biomass of different short tree layers.

In addition, we further fitted an SEM to interpret the casual effects and relative importance of stand age, soil nutrient regime, growing degree days, climate moisture index, species richness, and aboveground biomass of the tall tree layer on the species richness and aboveground biomass of the short tree, shrub, herb and liana layers (Table S1). The SEM in our study was based on the theoretical multivariate causes of plant diversity and ecosystem functioning in natural forests^36^. We explicitly defined two main paths: (1) the relationships between species richness and aboveground biomass within each layer, and (2) the effects of covariates, including GDD, SNR, CMI, SA and the tall tree layer, on the short tree layer. As recommended by Zhang *et al*.^7^, the GDD, SNR, SA, CMI and aboveground biomass across forest vegetation strata were transformed using the natural log scale. The standardized root mean square residual (SRMR) and goodness-of-fit index (GFI) were used to evaluate the goodness of fit of the SEMs with GFI values >0.95 and SRMR values <0.08. We report the standardized coefficient for each path from each component model. The total effects, as well as the direct and indirect standardized effects, were also calculated to strengthen the interpretation of our SEM results. The SEM analysis was implemented using the “lavaan” package in R 3.3.2^37^ (R development Core Team 2016).

## Electronic supplementary material

Supplementary Information
