# Supplementary material for: Positive relationship between species richness and aboveground biomass across forest strata in a primary Pinus kesiya forest
Source: Sci Rep. 2018 Feb 2;8:2227. doi: 10.1038/s41598-018-20165-y (PMC5797165; doi:10.1038/s41598-018-20165-y)
Supplement: Supplementary file 1 — Supplementary Information [file 41598_2018_20165_MOESM1_ESM.pdf]

Positive relationship between species richness and aboveground biomass across forest strata in a primary *Pinus kesiya* forest

Shuaifeng Li, Jiranrong Su, Xuedong Lang, Wande Liu, Guanglong Ou'

Supplementary information

Table S1. The aboveground biomass and species richness across forest vegetation strata as well as stand age, soil nutrient regime, growing degree days and climate moisture index in each plot. TTB, Tall tree aboveground biomass; STB, Short tree aboveground biomass; LB, Liana aboveground biomass; SB, Shrub aboveground biomass; HB, Herb aboveground biomass; TTR, Tall tree species richness; STR, Short tree species richness; LR, Liana species richness; SR, Shrub species richness; HR, Herb species richness; SR, Stand age; SNR, Soil nutrient regime; GDD, growing degree days; CMI, climate moisture index.

plot	TTB	STB	LB	SB	HB	TTR	STR	LR	SR	HR	SA	SNR	GDD	CMI
1	117	6.84	0	0	1.07	9	20	0	3	5	39.8	6.01	4579	1.31
2	173	7.35	0	0.02	0.76	8	22	0	1	4	40.5	5.91	4547	1.31
3	159	2.68	0.02	0	1.07	1	14	2	1	5	44.4	5.42	4701	1.31
4	50.4	5.92	0	0.01	0.75	1	15	0	1	4	37.6	5.4	4687	1.31
5	99.7	6.01	0	0.01	1.12	4	13	0	2	5	40.9	4.05	4656	1.31
6	116	15.6	0	0.01	1.35	3	15	0	2	6	41	5.45	4625	1.31
7	202	5.71	0	0	0.9	2	20	0	1	5	44.7	5.7	4666	1.31
8	122	6.84	0	0.01	0.94	4	18	0	1	4	45.2	4.99	4636	1.31
9	143	12.5	0	0	1.86	8	27	0	2	6	41.6	4.82	4698	1.31
10	105	18.5	0	0	0.96	13	28	0	2	4	45.1	5.74	4685	1.31
11	94.9	14.3	0.25	0.01	1.26	7	22	1	2	3	34.3	5.56	4682	1.31
12	70.6	13.9	0.19	0	1.42	9	25	1	2	6	33.8	5.5	4669	1.31
13	112	12.5	0.13	0.05	1.01	8	20	1	1	5	51.9	4.69	4872	1.29
14	252	12.6	0	0.02	0.48	11	18	0	3	5	56	4.86	4905	1.29
15	150	18.5	0	0.24	1.08	10	19	0	3	3	51.1	5.74	4738	1.29
16	468	6.61	0	0.03	0.81	6	17	0	4	3	51	5.01	4716	1.29
17	240	5.53	0.23	0.02	1.11	6	19	1	3	5	50.2	4.84	4732	1.29
18	183	4.54	0.06	0.02	1.84	8	19	1	3	7	51	3.89	4718	1.29
19	140	3.3	0	0.03	1.68	1	16	0	2	9	43.9	2.43	5100	1.33
20	140	2.91	0	0	2.04	2	17	1	2	8	59	3.35	5052	1.33
21	168	6.93	0.01	0.01	1.19	5	22	2	3	4	48.9	3.61	5042	1.33
22	116	10.5	0.01	0	0.53	3	27	1	1	5	44.5	2.95	5053	1.33
23	178	3.4	0	0	2.14	2	8	0	0	9	47.2	2.72	4842	1.33
24	172	3.07	0	0	2.9	3	8	0	0	7	46.2	3.65	4817	1.33
25	163	5.95	0.17	0	0.46	2	26	1	1	7	38.1	3.82	5149	1.32
26	150	5.84	0	0	2.21	5	22	0	1	8	38	3.56	5124	1.32
27	92.8	5.38	0	0.01	1.51	3	24	0	1	7	42	3.05	5199	1.32
28	74	7.33	0	0.02	0.23	4	23	0	3	4	45.1	3.32	5171	1.32
29	84.3	5.56	0	0.01	0.71	3	24	0	5	6	41.6	2.97	5167	1.32
30	144	2.26	0	0	0.9	2	21	0	1	5	40.9	2.29	5139	1.32
31	183	4.04	0	0	0.91	2	13	0	1	6	46.1	2.13	5251	1.27
32	193	3.95	0.03	0.01	0.87	3	21	1	2	5	41.4	2.79	5229	1.27

33	222	3.58	0	0.01	2.39	7	13	0	2	7	45.5	0.72	5214	1.27
34	128	2.39	0	0.02	1.13	2	15	0	4	5	41.8	0.42	5187	1.27
35	121	5.01	0	0	2.08	5	23	0	0	7	41.4	2.04	4967	1.28
36	137	3.97	0	1.15	0.84	9	15	0	2	4	48.4	6.17	4946	1.28
37	109	7.8	0	0.04	0.46	6	14	0	1	4	45.2	3.61	4975	1.36
38	136	3.38	0	0.33	0.79	5	12	0	1	4	39.4	3.54	4940	1.36
39	78.1	5.59	0	0	1.7	4	18	0	0	6	38.8	2.96	4676	1.36
40	113	7.73	0	0	1.03	5	19	0	1	5	38.2	2.6	4638	1.36
41	92.4	7	0	0	0.73	5	16	0	1	6	41.1	2.68	4676	1.36
42	106	6.81	0	0	0.72	2	15	0	1	5	38.3	3.27	4643	1.36
43	99	8.7	0.15	0	0.7	3	11	2	0	3	40	3.92	4772	1.37
44	111	11	0	0	0.51	5	11	0	0	3	43.6	2.36	4743	1.37
45	127	10.4	0.89	0	0.88	8	18	1	2	3	44.9	3.16	4772	1.37
46	90	13.2	0.41	0	0.78	5	14	2	0	4	41.4	5.25	4738	1.37
47	152	14.1	0	0	0.44	3	11	0	0	3	41.2	3.47	4743	1.38
48	47.7	15.4	0	0	0.42	4	8	0	0	3	30.5	3.21	4716	1.37
49	134	11.3	0	0	1.72	10	26	0	3	3	46.5	2.54	5271	1.29
50	230	11.7	0	0.01	1.22	6	26	0	4	2	44.2	2.88	5252	1.29
51	115	10.6	0	0.18	0.59	7	28	0	5	4	49.4	3.23	5297	1.29
52	292	6.32	0	0.17	1.23	9	18	0	5	3	49.8	1.93	5270	1.29
53	153	4.9	0	0	1.4	2	9	0	2	4	46.2	2.48	5290	1.29
54	209	4.08	0	0.3	1.88	2	13	0	2	4	56.2	4.07	5262	1.29
55	69.4	12.8	0	0.02	1.04	7	22	0	3	3	44.5	3.54	5304	1.29
56	155	18.5	0	0	0.68	7	19	1	1	6	47.9	3.43	5277	1.29
57	161	13.4	0.01	0	0.64	8	26	1	1	4	47.9	3.82	5291	1.29
58	196	14.6	0	0	2.3	8	23		2	4	45.8	4.36	5264	1.29
59	186	9.29	0.01	0	0.42	6	20	1	3	3	46.6	2.98	5525	1.33
60	143	14.1	0	0	1.19	7	24	1	3	5	41.7	2.61	5500	1.33
61	233	8.78	0	0.19	0.4	14	20	0	5	2	41.6	3.79	5066	1.29
62	255	11.6	0.2	0.08	0.92	8	24	1	3	2	46.1	3.22	5035	1.29
63	130	17.1	0.02	0.23	0.75	8	24	1	6	4	34.5	5.54	5060	1.29
64	86.9	20	0	0.1	1.64	6	24	0	5	6	33.8	4.48	5031	1.29
65	215	10.4	0.01	0.39	1.67	9	22	1	4	2	69.3	4.02	5070	1.29
66	191	13.9	0	0.87	0.97	8	19	1	3	5	47.5	3.35	5040	1.29
67	182	9.6	0.03	0.31	1.53	7	31	9	8	7	45.5	4.39	5052	1.29
69	35.6	8.88	0	0.01	0.64	8	30	4	4	4	33.6	4.8	5111	1.29
70	150	13.5	0.04	0.04	2.17	12	26	6	4	7	41.2	5.1	5109	1.29
71	79.5	14	0.08	0.21	0.86	8	24	1	2	3	28.8	6.45	5191	1.29
72	113	17	0.1	0	0.94	8	22	1	1	3	43.5	6.45	5191	1.29
73	94.2	15.7	0	0	1.2	9	19	0	0	3	34.3	4.76	5200	1.29
74	46.4	12.3	0.19	0.02	0.95	6	19	1	1	3	33.5	4.76	5200	1.29
75	148	17.8	0.07	0.03	0.24	10	18	2	1	2	42.1	5	5173	1.29
76	101	15.5	0.02	0.01	0.65	8	19	1	1	2	44.4	6.34	5169	1.28

77	114	8.59	0.04	0.01	0.37	9	16	1	1	2	38.2	5.29	5169	1.28
78	108	14.9	0.02	0.05	0.07	6	15	1	1	2	45.7	8.73	5114	1.29
79	102	17.9	0	0.41	0.33	7	11	0	1	2	41.1	7.43	5157	1.29
80	141	12.2	0.04	0.09	0.07	14	16	1	2	1	43.9	7.77	5114	1.29
81	110	9.81	0.01	0.04	0.26	9	20	1	2	1	41.6	7.77	5114	1.29
82	116	14	0	0.33	2.25	8	18	0	2	5	42.2	7.59	5141	1.29
83	139	10.2	0.07	0.03	0.1	8	22	1	2	1	35.5	8.81	5141	1.29
84	171	14.9	0	0.04	0.1	11	16	0	3	1	42.2	8.81	5139	1.29
85	153	12.2	0.02	0.08	0.5	16	27	3	2	2	47.5	6.03	5111	1.29
86	172	3.46	0.67	0.02	0.58	11	17	2	1	2		5.68	5108	1.29
87	123	8.73	0.13	0.03	1.59	12	17	2	1	3	52	4.13	5108	1.29
88	159	12.5	0.33	0.08	0.51	13	22	3	2	2	45.1	4.96	5090	1.29
89	335	7.78	0.04	0.01	1.99	11	22	1	1	4	46.7	0.91	5146	1.29
90	165	7.61	0.03	0.02	0.09	9	21	1	2	1	39.9	6.79	5125	1.29
91	259	5.34	0.02	0.01	0.37	10	19	1	1	2	58.5	6.48	5134	1.29
92	152	9.25	0.05	0.01	0.46	11	18	1	1	2	35.5	6.11	5155	1.29
93	159	14.1	0.15	0	0.34	9	17	2	0	2	42.4	7.45	5132	1.29
94	142	10.9	0.13	0	0.64	10	21	1	0	4	42.2	6.81	5132	1.29
95	155	16	3.73	0.55	0.32	11	29	6	5	2	49	6.64	5183	1.29
96	111	12.5	3.61	0.11	1.03	15	19	3	3	3	39.2	5.02	5167	1.29
97	236	9.87	0.57	0.24	1.68	12	27	5	4	6	51.3	7.33	5178	1.29
98	166	8.5	0.46	0.21	1.74	13	26	3	4	4	39.3	4.65	5178	1.29
99	164	5.79	0.43	0.04	1.02	13	19	2	2	4	47.5	5.06	5176	1.29
100	157	9.74	0	0.02	0.5	11	23	0	1	3	43.3	5.06	5176	1.29
101	198	8.91	0.04	0	0.31	9	20	1	1	2	58.1	5.23	5160	1.29
102	485	16.1	0.14	0.03	0.84	10	19	4	2	5	59.4	5.23	5160	1.29
103	296	14.9	0	0	0.56	7	20	0	0	3	60.7	5.18	5162	1.29
104	191	7.28	0.23	0.02	0.51	11	20	1	1	3	55.5	5.18	5162	1.29
105	415	14.1	0.02	0.01	1.14	7	18	1	1	5	48.8	6.88	5162	1.29
106	294	12.8	0.04	0.28	0.39	14	18	1	1	2	53.9	6.88	5149	1.29
107	327	11.1	0	0.05	0.3	11	17	0	2	2	57.7	4.5	5149	1.29
108	98.7	17.1	0.08	0.01	1.04	9	22	3	2	4	61.1	4.5	5152	1.29
109	169	11.1	0.9	0	0.44	10	20	3	0	2	67.8	4.84	5152	1.29
110	392	9.52	0	0.03	0.77	10	26	0	1	4	51.6	6.24	5117	1.29
111	256	8.93	0.21	0.02	1.14	9	24	3	1	4	51.8	6.01	5117	1.29
112	355	8.34	0.32	0.07	0.64	6	19	2	3	3	53.6	6.43	5117	1.29
113	428	8.38	0.01	0.36	0.44	6	24	1	3	4	71.3	6.08	5117	1.29
114	458	7.08	0	0	0.19	6	20	0	0	3	58	5.98	5111	1.29
115	255	7.98	0.55	0.41	0.74	10	19	5	2	3	60.8	5.98	5105	1.29
116	295	9.47	1.49	0.58	0.18	14	28	7	6	1	59.2	6.36	5166	1.28
117	321	5.65	0.22	0.08	0.92	15	30	3	4	4	64	6.02	5169	1.29
118	387	12.7	0.11	0.11	0.8	8	27	4	4	4	56	6.39	5169	1.29
120	462	10.9	0.04	0.07	0.71	11	30	2	3	4	63.1	5.66	5180	1.29

121	271	11.6	0.12	0.01	0.46	9	31	2	2	3	52.7	5.66	5178	1.29
122	497	6.97	0.67	0.04	1.28	9	21	4	4	4	67.8	5.66	5178	1.29
123	287	12.1	0.01	0.02	0.78	10	32	1	4	4	70.9	6.99	5199	1.29
124	257	13.5	0.21	0.52	1.05	10	26	1	4	3	60.5	7.63	5183	1.29
125	442	8.19	0.11	0.01	1.5	8	27	3	2	6	55.1	6.99	5183	1.29
126	235	9.07	0	0.11	0.44	9	20	0	3	3	62.3	5.87	5181	1.28
127	506	5.72	0	0.1	2.77	9	25	0	6	8	63.9	7.03	5192	1.29
128	274	7.85	0.1	0.01	3.53	9	24	2	3	11	66.1	7.03	5192	1.29
129	322	8.76	0.07	0	0.53	8	25	1	0	4	70.5	6.09	5226	1.29
130	163	10.4	0.01	0.03	0.66	7	26	1	2	4	63.2	4.92	5226	1.29
131	278	10.3	0	0.07	0.31	10	23	0	4	3	54.7	5.13	5206	1.29
132	451	9.32	0.06	0.02	0.99	10	24	4	3	4	64.5	4.9	5204	1.28
133	148	9.79	0.01	0.02	1.67	9	29	1	2	6	52.3	4.81	5220	1.28
134	459	9.26	0.33	0.51	1.68	7	37	2	2	9	58.9	4.62	5206	1.28
135	196	9.63	0.33	0.02	1.56	9	40	6	4	8	64.7	0.77	5206	1.28
136	323	9.69	0.13	0.08	0.24	8	31	3	4	1	74.4	3.97	5198	1.29
137	322	9.08	0.29	0.14	0.89	10	32	2	5	3	65.3	3.97	5198	1.29
138	333	8.05	0	0.07	0.85	8	30	0	4	4	54.2	4.31	5184	1.28
139	436	8.09	0.17	0.05	0.8	8	30	2	5	4	77.2	4.31	5184	1.28
140	393	8.4	0	0.02	1.46	7	26	0	2	6	66.7	4.65	5184	1.29
141	341	7.75	0	0.04	2.27	11	24	0	1	9	59.8	4.65	5184	1.29
142	406	7.49	0.5	0.31	0.94	13	31	1	5	3	55.9	5.85	5223	1.29
143	339	10.9	0.27	0.3	1.79	11	24	2	6	6	64.2	4.06	5223	1.29
144	203	10.1	0.69	0.03	0.94	9	32	1	2	4	46.5	4.06	5196	1.29
145	226	9.69	0.96	0.13	1	13	29	2	3	4	49	3.75	5196	1.29
146	466	7.85	0.48	0.14	0.91	9	37	6	7	3	62.7	3.75	5197	1.29
147	216	10.4	0.43	0.12	0.88	8	30	4	5	5	55.2	4.87	5197	1.29
148	296	6.77	0.63	0.04	1.59	10	22	4	4	6	49.2	4.87	5213	1.29
149	268	8.65	0.26	0.09	2.24	9	26	4	6	7	53.8	3.75	5213	1.29
150	222	2.49	0.06	0.01	1.82	6	21	1	2	5	46.9	3.75	5167	1.29
151	384	3.24	0.02	0.02	1.24	6	22	1	1	5	59.3	3.58	5167	1.29
152	230	3.7	0.05	0	1.17	8	18	2	2	4	51.7	4.09	5139	1.29
153	267	4.76	0.01	0	1.8	5	21	1	1	7	51	4.23	5139	1.29
154	309	6.23	0.01	0.05	1.36	7	22	2	2	4	56.6	4.23	5144	1.29
155	217	2.05	0.06	0.02	1	5	24	1	3	4	55	5.35	5144	1.29
156	395	4.52	0.03	0	1.69	6	17	1	0	5	52.8	3.98	5159	1.29
157	348	7.79	0.32	0	2.3	9	20	1	0	7	64.4	3.98	5167	1.29
158	456	7.58	0.27	0	1.71	6	13	1	0	6	59.4	3.91	5167	1.29
159	243	7.63	0	0	1.53	7	15	0	1	4	61	3.91	5145	1.29
160	381	12.4	0.47	0	1.43	10	18	1	0	4	61.8	4.71	5145	1.29
161	290	9.65	0	0	1.64	10	18	0	0	6	55.4	4.71	5143	1.29
162	311	8.47	0.16	0	1.11	12	19	4	0	4	58.4	4.08	5143	1.29
163	285	11.5	0.03	0.08	1.41	13	20	1	2	4	68.4	4.08	5160	1.29

164	176	7.96	0.21	0	1.58	12	18	2	0	5	58.8	4	5160	1.29
165	266	7.39	0.11	0	1.17	8	20	1	0	5	52.3	4	5160	1.29
166	355	9.83	0.06	0.02	1.24	11	20	1	2	4	51.3	4.31	5188	1.29
167	394	9.15	0	0.01	1.13	8	21	0	1	5	57.4	5.19	5188	1.29
168	325	11	0.25	1.26	0.88	8	23	1	3	5	65.2	5.19	5186	1.29
169	214	7.36	0.05	0.38	0.95	5	27	1	5	4	58.5	4.49	5186	1.29
170	398	10.4	0.03	0.02	0.66	5	24	1	1	4	54	3.51	5197	1.29
171	289	8.57	0.06	0.02	0.82	5	27	1	3	4	55.5	4.6	5199	1.29
172	139	9.75	0.27	0.15	1.67	10	26	3	5	7	59	4.31	5199	1.29
